# The protective effects of Chromofungin in oligomeric amyloid β_42_ (Aβ_42_)-induced toxicity in neurons in Alzheimer’s disease

**DOI:** 10.18632/aging.205865

**Published:** 2024-05-24

**Authors:** Qingwei Li, Ji Sun, Qin Ran, Ziming Liu, Jinmei Chen

**Affiliations:** 1 Department of Psychiatry, Tongji Hospital, School of Medicine, Tongji University, Shanghai 200065, China; 2 Shanghai Mental Health Center, Shanghai Jiao Tong University School of Medicine, Shanghai 200030, China; 3 Department of Neurology, Shanghai Ninth People’s Hospital, Shanghai Jiao Tong University School of Medicine, Shanghai 201999, China

**Keywords:** Alzheimer’s disease, Chromofungin, senescence, oxidative stress, oligomeric Aβ_42_

## Abstract

Oligomeric Aβ_42_ is considered to play a harmful role in the pathophysiology of Alzheimer’s disease (AD). Prolonged exposure to oligomeric Aβ_42_ could induce neuronal damage including cellular senescence. Amelioration of Aβ_42_-induced cellular senescence has been considered as a promising strategy for the treatment of AD. Chromofungin, a chromogranin A-derived peptide, has displayed various biological functions in different types of cells and tissues. However, the effects of Chromofungin on oligomeric Aβ_42_-induced cellular senescence have not been previously reported. In the current study, we report a novel function of Chromofungin by showing that treatment with Chromofungin could ameliorate Aβ_42_-induced neurotoxicity in M17 neuronal cells. The Cell Counting Kit-8 (CCK-8) assay and the lactate dehydrogenase (LDH) release experiments revealed that 0.5 and 1 mM are the optimal concentrations of Chromofungin for cell culture in M17 cells. Challenging with oligomeric Aβ_42_ (5 μM) for 7 and 14 days led to a significant decrease in telomerase activity, which was rescued by Chromofungin dose-dependently. Additionally, the senescence-associated β-galactosidase (SA-β-gal) staining assay demonstrated that Chromofungin mitigated oligomeric Aβ_42_-induced cellular senescence. Correspondingly, treatment with Chromofungin reversed the gene expression of human telomerase reverse transcriptase (hTERT), telomeric repeat-binding factor 2 (TERF2), and p21 against oligomeric Aβ_42_ in M17 neurons. Interestingly, Chromofungin attenuated oligomeric Aβ_42_-induced oxidative stress (OS) in M17 cells by reducing the production of intracellular reactive oxygen species (ROS) but increasing the levels of intracellular superoxide dismutase (SOD). Importantly, the presence of Chromofungin reduced the expression of cyclooxygenase2 (COX-2) as well as the generation of prostaglandin E_2_ (PGE_2_). Transduction with Ad-COX-2 impaired the effects of Chromofungin on telomerase activity and the profile of cellular senescence. Our findings suggest that Chromofungin might act as a potential agent for the treatment of AD.

## INTRODUCTION

Alzheimer’s disease (AD) is a progressive neurodegenerative disorder and one of the most common forms of dementia in the elderly [1]. The patient’s memory gradually deteriorates, making it difficult to remember new information and even familiar people and things. Additionally, cognitive function declines, making it challenging for patients to perform simple activities of daily living such as dressing and eating independently. Furthermore, patients exhibit significant changes in behavior, including emotional instability, irritability, aggression, and getting lost. In the most severe cases, patients may completely lose their ability to take care of themselves and require assistance from others, severely impacting their quality of life [2–4]. Recent studies have shown a close correlation between the development of AD and the abnormal aggregation of β-amyloid 42 (Aβ_42_) [5, 6]. Aβ_42_ is a peptide consisting of 42 amino acids generated by the cleavage of amyloid precursor protein (APP) by enzymes [7]. Under normal circumstances, Aβ_42_ is cleared without accumulating in the brain. However, in AD patients, Aβ_42_ abnormally aggregates to form amyloid plaques, which are one of the main characteristics of the disease [8, 9]. Research has found that the aggregation of Aβ_42_ triggers the process of cellular senescence, which refers to the gradual deterioration of cell function and structure, leading to decreased metabolic activity and functional abnormalities [10]. Cellular senescence is closely associated with the development of AD, primarily manifested by the degeneration and death of brain neurons [11]. The abnormal aggregation of Aβ leads to increased oxidative stress (OS), impaired mitochondrial function, and enhanced inflammatory responses, thereby initiating the process of cellular senescence [12]. Cellular senescence not only causes the death of brain neurons but also disrupts normal communication and signal transmission between neurons, ultimately resulting in the loss of memory and cognitive function [13]. Understanding the correlation between Aβ_42_ and cellular senescence is of significant importance for treating AD. By intervening in the abnormal aggregation of Aβ_42_ and the process of cellular senescence, it is possible to halt the progression of the disease and alleviate the decline in cognition and abnormal behavior in patients.

Chromofungin, a short CgA N-terminal peptide with an α-helical structure, is produced by proteolytic cleavage through hormone-stimulating convertases [14]. It serves as the major constituent of secretory granules in neuroendocrine cells [15], exhibiting anti-inflammatory and antimicrobial activities. Moreover, Chromofungin is a bioactive substance with antifungal activity, which is a class of peptides produced by fungi, possessing a broad spectrum of antifungal effects, capable of inhibiting the growth and reproduction of various fungi [16, 17]. Chromofungin has a unique molecular structure and bioactivity, and making it a potential natural antifungal drug [16]. Chromofungin peptide is reported to block the growth and reproduction of fungi by interfering with the integrity of fungal cell membranes and the synthesis of cell walls [18]. Furthermore, Chromofungin also functions in regulating the immune system and antioxidant activity, contributing to enhancing the body's defense against fungal infections [19]. However, the function of Chromofungin in neurodegenerative disorder diseases remains unclear. Herein, the influence of Chromofungin on the cell senescence of neurons was tested to explore the potential pharmacological effects of Chromofungin against AD.

## MATERIALS AND METHODS

### Preparation of oligomeric Aβ_42_

The Aβ_42_ peptide (Invitrogen, USA) was initially dissolved in 100% hexafluoroisopropanol (HFIP) at a concentration of 1 mM. Subsequently, the HFIP was eliminated via vacuum evaporation. The peptide was then reconstituted in dimethyl sulfoxide (DMSO) at a concentration of 5 mM. F12 (phenol red-free) culture medium (Invitrogen, USA) was introduced to attain a final peptide concentration of 100 μM, followed by incubation at 4°C for 24 h. The resulting solution underwent centrifugation at 13000 r.p.m. for 20 min, and the resulting supernatant was preserved at −20°C until further use.

### Cell culture, transduction, and treatment

M17 neuronal cells were obtained from American Type Culture Collection (ATCC) (USA) and cultured in OptiMEM medium supplemented with 5% fetal bovine serum (FBS) at 37°C/5% CO_2_. To overexpress COX-2 in M17 neuronal cells, cells were transduced with the adenovirus containing COX-2-overexpressed vector (Ad-COX-2) for two days, which were identified using the Western blotting assay.

### Cell Counting Kit-8 (CCK-8) assay

The 96-well tray was positioned in a constant temperature chamber for a duration of 24 hours. The medium in every well was removed, and a pre-blended CCK-8 solution was introduced to each well, ensuring gentle agitation for complete integration. Subsequently, the 96-well tray was returned to the constant temperature incubator to facilitate thorough staining. A microplate reader (Molecular Devices, USA) was utilized to measure the optical density at 450 nm for each well, and the results were duly documented. The cell viability was determined by analyzing the recorded optical density values.

### Lactate dehydrogenase (LDH) release assay

A volume of 30 μL of the supernatant was obtained to assess LDH release, utilizing a commercially available kit (Abcam, USA). Subsequently, the optical density at 450 nm was measured using a microplate reader (Molecular Devices, USA) to determine the percentage of LDH release.

### Determination of telomerase activity

The experiment was performed using the TRAP-EUSA kit. Cells were collected and washed with PBS, and then resuspended in 200 μL lysis buffer. After incubation on ice for 30 min, samples were centrifuged at 16,000 × g for 20 min at 4°C. The supernatant was collected for UV protein quantification. Approximately 5 μg of cell lysate was added to each tube, along with 25 μL of the mixture, 5 μL of internal standard primer, and water without DNAase to make a total volume of 50 μL. The reaction mixture was incubated at 25°C for 30 minutes, followed by heating at 94°C for 5 min to inactivate the reaction. PCR was then performed. Samples and internal control PCR products (2.5 μL each) were dispensed onto a 96-well plate. Blank lysis buffer and inactivated control samples were included as controls. Each well was added with 10 μL denaturing solution and incubated for 10 min. Samples were divided into two, and 100 μL of hybridization buffer T or 100 μL of hybridization buffer IS was added to each half. After mixing, samples were transferred to a pre-treated 96-well plate, sealed, and incubated at 37°C with shaking at 300 rpm for 2 h. The hybridization solution was then removed, and 100 μL of Anti-DIG-HRP working solution was added. The plate was centrifuged at 300 rpm and cooled for 30 min. After discarding the hybridization solution, 100 μL of TMB substrate solution was added, and the plate was incubated with shaking at 300 rpm for 20 min. Finally, 100 μL of stop solution was added, and the absorbance was measured at 450 nm within 30 min to calculate the relative activity of the telomerase.

### Senescence-associated β-galactosidase (SA-β-gal) staining

SA-β-gal staining was performed using an SA-β-gal staining kit (Cell Signaling Technology, USA). Briefly, M17 neuronal cells were plated onto a 12-well culture dish and allowed to adhere for 24 h. Following treatments, the culture medium was removed and cells were washed with PBS and then fixed using a 1× Fixative solution for 15 min. A staining solution for β-Galactosidase was prepared and adjusted to pH 6.0 using a pH meter. The staining solution was added to cells and incubated at 37°C overnight without the presence of CO_2_ in an incubator. After the removal of the staining solution, cells were washed with PBS and then covered with a water-based sealant and stored at 4°C for observation using an upright microscope (Leica, Germany). The SA-β-gal positive cell ratio was counted in three random fields for every culture dish in a blinded manner [20].

### Dichloro-dihydro-fluorescein diacetate (DCFH-DA) staining assay

After receiving the appropriate treatments, the growth medium was removed. The cells were then cultured in a serum-free medium supplemented with a 5 μmol/L DCFH-DA probe at a temperature of 37°C for a duration of 20 min. The medium was removed and replaced with 1 mL of PBS. The intensity of fluorescence was visualized using a fluorescence microscope (Leica, Germany).

### Measurement of superoxide dismutase (SOD)

The SOD activity in the supernatant of M17 neuronal cells was assessed using the colorimetric method with a commercial kit (Abcam, USA), strictly following the instructions provided with the kit.

### Real-time polymerase chain reaction (PCR)

Cellular samples were thoroughly lysed using Trizol reagent to extract the entire RNA. The concentration and purity of the RNA samples were assessed using a UV-visible spectrophotometer, followed by reverse transcription to generate cDNA. The resultant cDNA samples were amplified utilizing a RT-PCR instrument (Agilent, USA). GAPDH was employed as the standard reference gene, and gene levels were determined using the 2^−ΔΔCt^ method. Primer sequences are shown in Table 1.

**Table 1:** Primer sequences.

**Primer**	**Sense 5′–3′**	**Anti-sense 5′–3′**
COX-2	TGCATTCTTTGCCCAGCACT	AAAGGCGCAGTTTACGCTGT
hTERT	CGGAAGAGTGTCTGGAGCAA	GGATGAAGCGGAGTCTGGA
TERF2	GTACCCAAAGGCAAGTGGAA	TGACCCACTCGCTTTCTTCT
p21	CCTCTTCGGCCCGGTGGAC	CCGTTTTCGACCCTGAGAG
GAPDH	TGAAGGTCGGTGTGAACGGATTTGGC	CATGTAGGCCATGAGGTCCACCAC

### Western blotting assay

The whole cellular protein was obtained from cells employing radioimmunoprecipitation assay (RIPA) lysis solution. The concentration of the protein was determined utilizing the bicinchoninic acid (BCA) method. The protein was segregated by gel electrophoresis and transferred to a polyvinylidene fluoride (PVDF) membrane, which was subsequently blocked. The membrane was exposed to primary antibodies COX-2 (1:1000) and β-actin (1:2000, Abcam, USA) overnight. It was then rinsed and incubated with a secondary antibody (1:4000, Abcam, USA) for 120 min. The membrane was then cultured with electrochemiluminescence (ECL) solution, and images were captured utilizing a gel imaging analysis system. The grayscale analysis was performed employing the software ImageJ. The grayscale analysis of target bands was conducted using the software ImageJ. Briefly, target bands were selected and the background was eliminated. Subsequently, the integrated optical density of each band was calculated and exported for further analysis.

### Enzyme-linked immunosorbent assay (ELISA)

Production of PGE_2_ was measured using a commercial kit (Abcam, USA). The dish was initially coated with the antigen and subsequently blocked. The specimen and a positive control were then introduced to the dish and incubated for 1 h. Following a thorough wash to eliminate any unbound substances, an enzyme-linked conjugate was introduced to amplify the signal. Then, a substrate solution was added, and the reaction was allowed to progress for 20 min. The absorbance for each sample was measured at 450 nm with a spectrophotometer.

### Statistical analysis

The experimental results were presented as mean ± standard deviation and analyzed utilizing SPSS18.0 statistical software. One-way analysis of variance (ANOVA) was used for multiple group comparisons. A significance level of *P* < 0.05 was considered to indicate a significant distinction.

### Data availability

The data are available upon reasonable request from the corresponding author.

## RESULTS

### The cytotoxicity of Chromofungin in M17 neuronal cells

**Figure 1:**
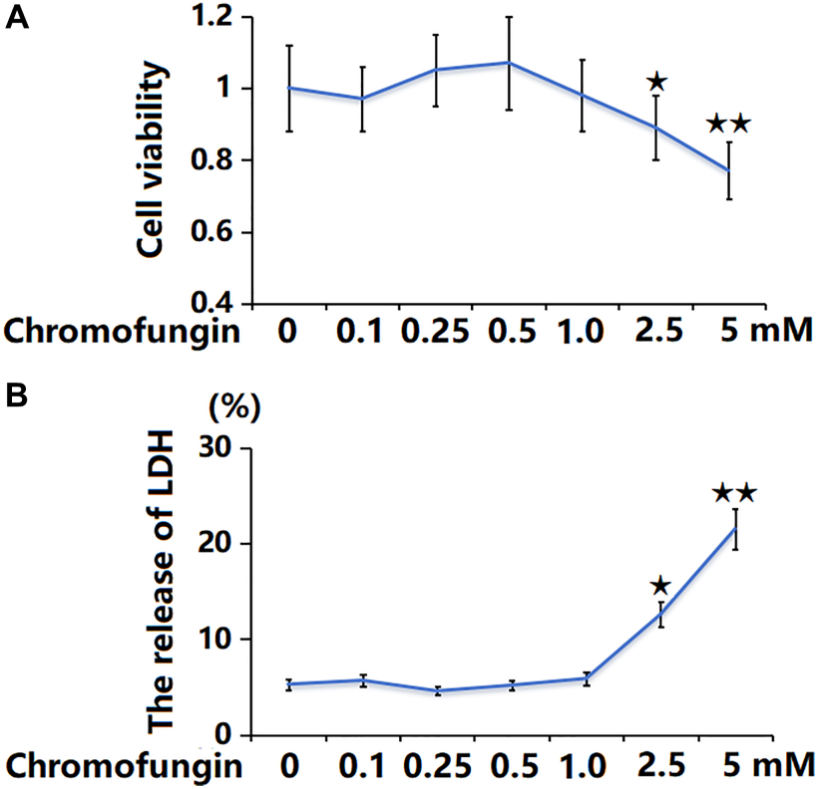
**The cytotoxicity of Chromofungin in M17 neuronal cells.** Cells were stimulated with Chromofungin at the concentrations of 0.1, 0.25, 0.5, 1, 2.5, and 5 mM for 48 hours. (**A**) Cell viability of M17 neuronal cells was measured using the CCK-8 assay; (**B**) The release of LDH was measured using a kit (*n* = 6, ^*, **^P < 0.05, 0.01 vs. vehicle group).

To determine the concentration of Chromofungin, M17 neuronal cells were stimulated with Chromofungin at the concentrations of 0.1, 0.25, 0.5, 1, 2.5, and 5 mM for 48 h. The cell viability was notably reduced to 89% and 77% by 2.5 and 5 mM Chromofungin, respectively (Figure 1A). Additionally, the LDH release was significantly increased to 12.6% and 21.5% by 2.5 and 5 mM Chromofungin, respectively (Figure 1B). Consequently, 0.5 and 1 mM Chromofungin were applied in subsequent experiments.

### Chromofungin restored telomerase activity against oligomeric Aβ_42_ in M17 cells

**Figure 2:**
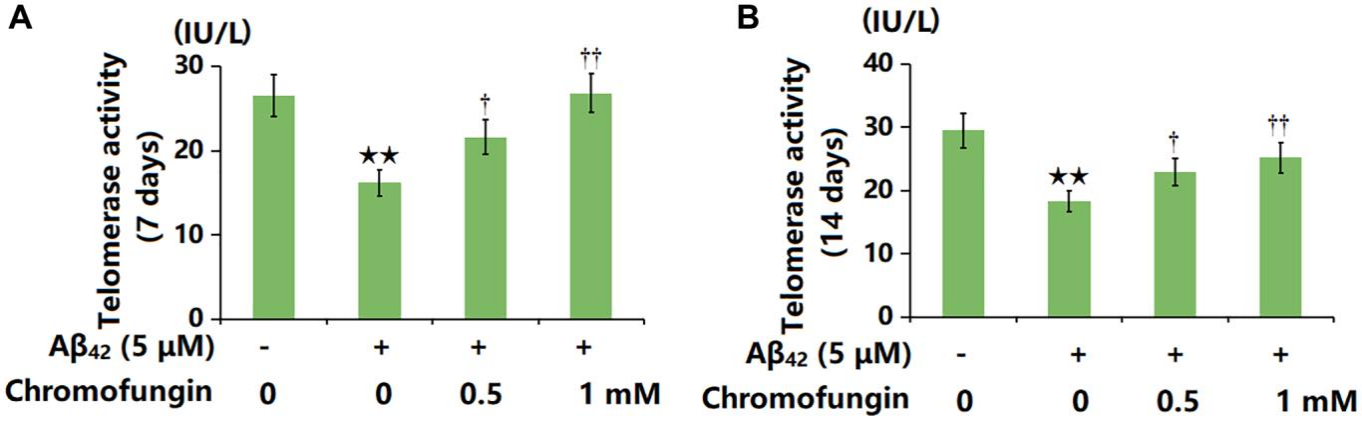
**Chromofungin restored telomerase activity against oligomeric Aβ_42_ in M17 cells.** Cells were incubated with oligomeric Aβ_42_ (5 μM) with or without Chromofungin (0.5, 1 mM). (**A**) Telomerase activity at 7 days after incubation; **(B**) Telomerase activity at 14 days after incubation (*n* = 6, ^**^P < 0.01 vs. vehicle group; ^†, ††^*P* < 0.05, 0.01 vs. Aβ_42_ group).

M17 cells were incubated with oligomeric Aβ_42_ (5 μM) with or without Chromofungin (0.5, 1 mM). After a 7-day incubation, the telomerase activity was notably reduced from 26.5 to 16.2 IU/L by Aβ_42_ and markedly elevated to 21.6 and 23.8 IU/L by 0.5 and 1 mM Chromofungin, respectively (Figure 2A). Moreover, after a 14-day incubation, the telomerase activity values in the control, Aβ_42_, 0.5 mM Chromofungin, and 1 mM Chromofungin groups were 29.5, 18.3, 22.9, and 25.2 IU/L, respectively (Figure 2B).

### Chromofungin attenuated oligomeric Aβ_42_-induced cellular senescence of M17 neuronal cells

**Figure 3:**
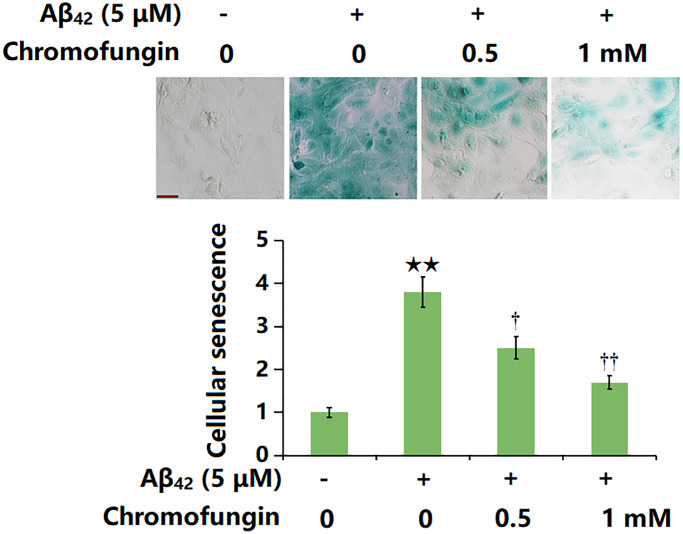
**Chromofungin attenuated oligomeric Aβ_42_-induced cellular senescence of M17 neuronal cells.** Cells were incubated with oligomeric Aβ_42_ (5 μM) with or without Chromofungin (0.5, 1 mM). Cellular senescence was examined using SA-β-gal staining at day 14. Scale bar, 50 μm (*n* = 6, ^**^P < 0.01 vs. vehicle group; ^†, ††^*P* < 0.05, 0.01 vs. Aβ_42_ group).

SA-β-gal is widely regarded as the most commonly used biomarker for senescent and aging cells. The representative SA-β-gal staining and quantification are shown in Figure 3. Following a 14-day incubation, the proportion of SA-β-gal positive staining cells was markedly increased by Aβ_42_ stimulation, which was remarkably repressed by 0.5 and 1 mM Chromofungin.

### Chromofungin affected the expressions of hTERT, TERF2 and p21 in oligomeric Aβ_42_-challenged M17 neuronal cells

**Figure 4:**
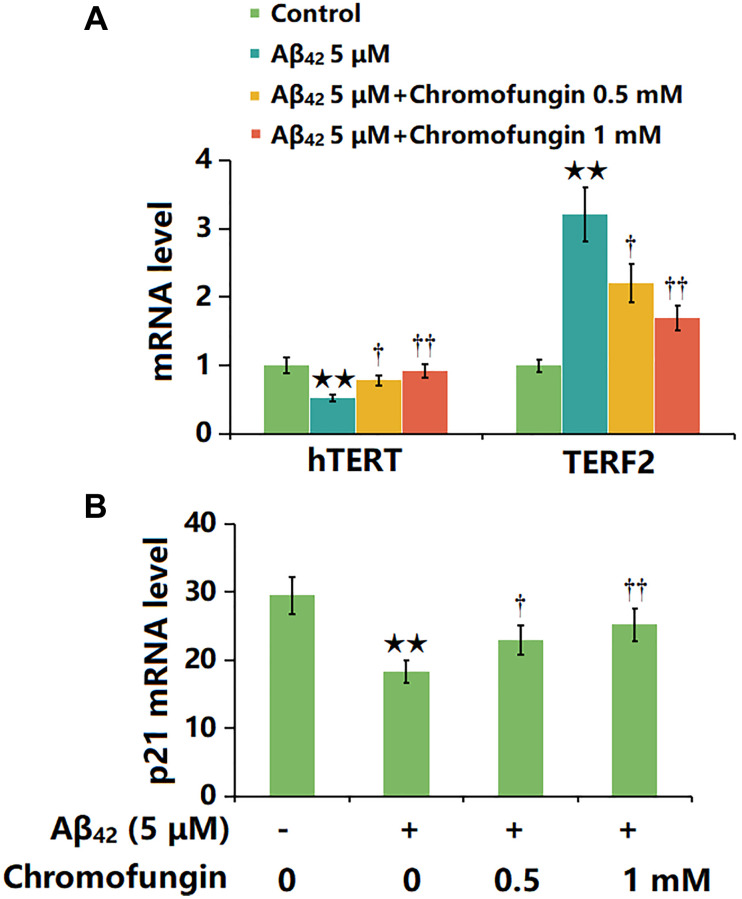
**Chromofungin affected the expressions of hTERT and TERF2 in oligomeric Aβ_42_-challenged M17 neuronal cells.** Cells were stimulated with oligomeric Aβ_42_ (5 μM) with or without Chromofungin (0.5, 1 mM) for 24 hours. (**A**) mRNA expression of hTERT; (**C**) mRNA of p21 (*n* = 6, ^**^P < 0.01 vs. vehicle group; ^†, ††^*P* < 0.05, 0.01 vs. Aβ_42_ group).

M17 cells were stimulated with oligomeric Aβ_42_ (5 μM) with or without Chromofungin (0.5, 1 mM) for 24 h, followed by the detection of senescence-related gene levels. The hTERT level was sharply reduced by Aβ_42_ but considerably elevated by 0.5 and 1 mM Chromofungin, while the TERF2 expression was markedly increased by Aβ_42_ but remarkably reduced by 0.5 and 1 mM Chromofungin (Figure 4A). Additionally, the highly upregulated p21 observed in Aβ_42_-challenged M17 neuronal cells was markedly downregulated by 0.5 and 1 mM Chromofungin (Figure 4B).

### Chromofungin ameliorated oligomeric Aβ_42_-induced OS in M17 neuronal cells

**Figure 5:**
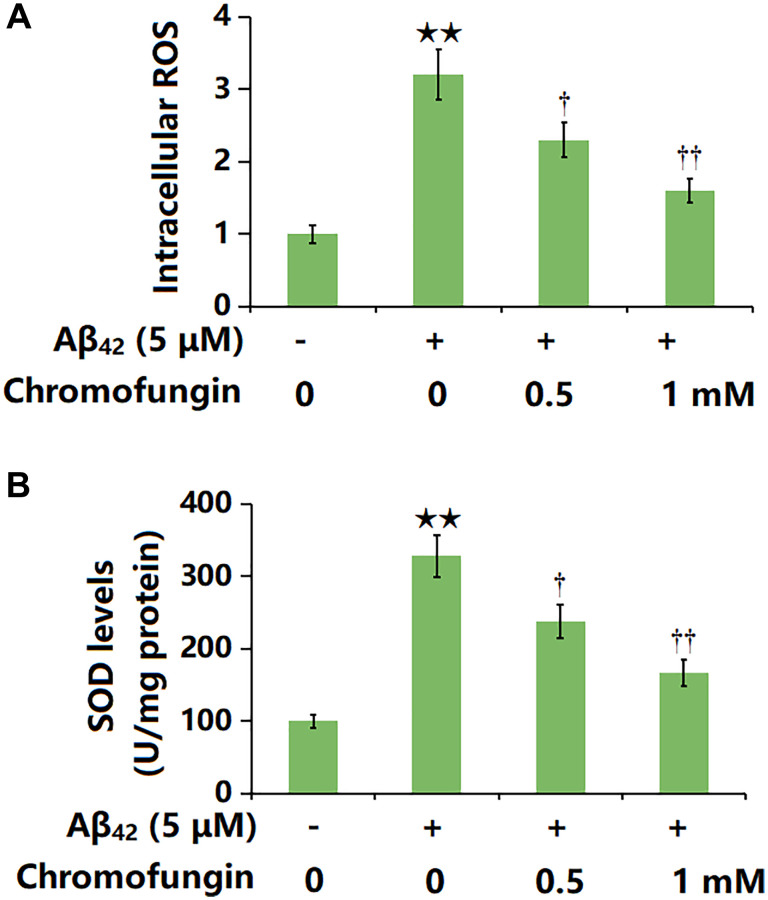
**Chromofungin ameliorated oligomeric Aβ_42_-induced oxidative stress in M17 neuronal cells.** Cells were stimulated with oligomeric Aβ_42_ (5 μM) with or without Chromofungin (0.5, 1 mM) for 24 hours. (**A**) Intracellular ROS was measured using DCFH-DA staining; (**B**) The levels of SOD (*n* = 6, ^**^P < 0.01 vs. vehicle group; ^†, ††^*P* < 0.05, 0.01 vs. Aβ_42_ group).

The intracellular ROS levels in M17 neuronal cells were notably increased by Aβ_42_ and greatly reduced by 0.5 and 1 mM Chromofungin (Figure 5A). Furthermore, the SOD activity was markedly repressed by Aβ_42_, which was greatly reversed by 0.5 and 1 mM Chromofungin (Figure 5B).

### Chromofungin decreased the levels of COX-2 and the generation of PGE_2_ against oligomeric Aβ_42_ in M17 neuronal cells

**Figure 6:**
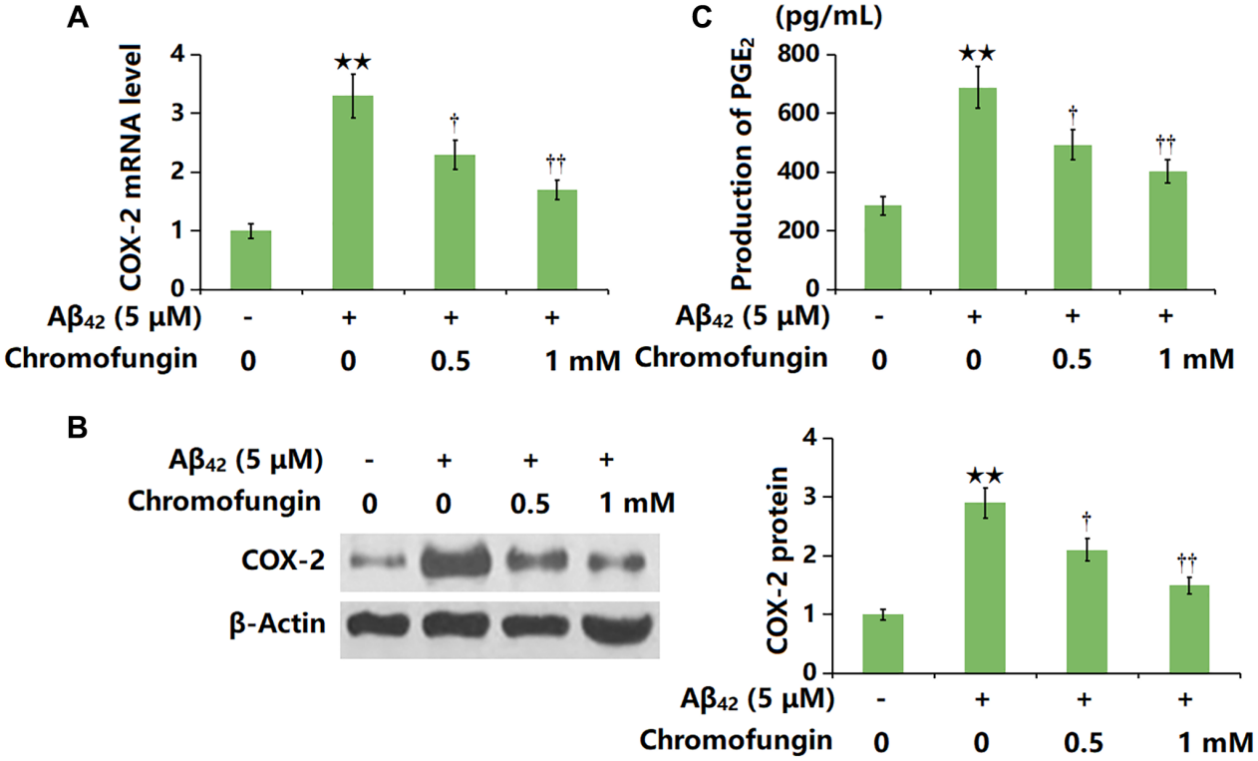
**Chromofungin decreased the levels of COX-2 and the generation of PGE_2_ against oligomeric Aβ_42_ in M17 neuronal cells.** Cells were incubated with oligomeric Aβ_42_ (5 μM) with or without Chromofungin (0.5, 1 mM) for 24 hours. (**A**) mRNA of COX-2 as measured by real-time PCR; (**B**) Protein expression of COX-2 as measured by western blot; (**C**) Production of PGE_2_ as measured by ELISA (*n* = 6, ^**^P < 0.01 vs. vehicle group; ^†, ††^*P* < 0.05, 0.01 vs. Aβ_42_ group).

COX-2 is reported to be a critical inducer of cell senescence [21]. COX-2 levels in Aβ_42_-stimulated M17 neuronal cells were sharply increased, but markedly repressed by 0.5 and 1 mM Chromofungin (Figure 6A, 6B). Moreover, the production of PGE_2_ in Aβ_42_-stimulated M17 neuronal cells was elevated from 286.2 to 688.5 pg/mL but reduced to 493.3 and 402.9 pg/mL by 0.5 and 1 mM Chromofungin, respectively (Figure 6C).

### Overexpression of COX-2 impaired the beneficial function of Chromofungin against oligomeric Aβ_42_-induced cellular senescence in M17 neuronal cells

**Figure 7:**
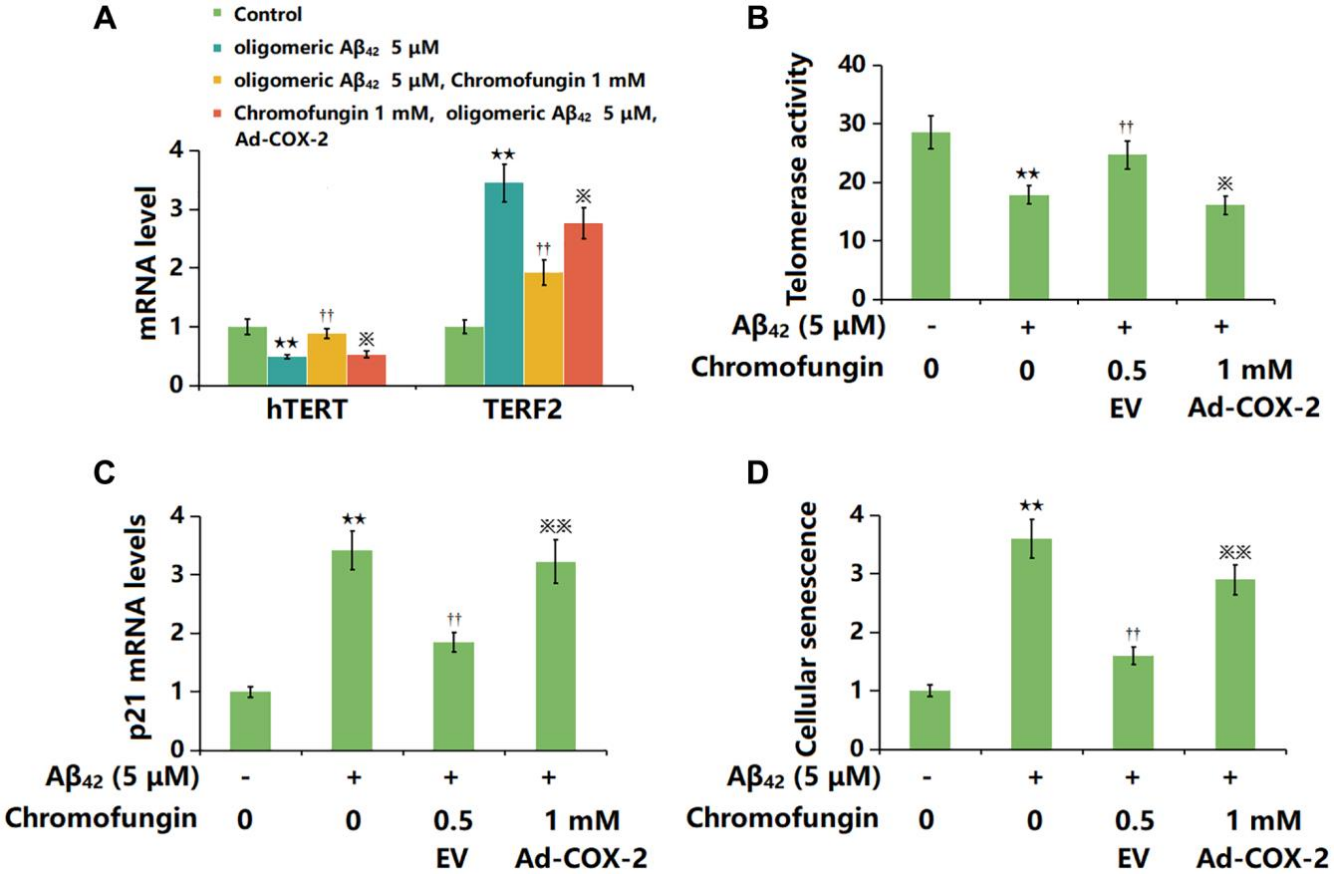
**Overexpression of COX-2 impaired the beneficial function of Chromofungin against oligomeric Aβ_42_-induced cellular senescence in M17 neuronal cells.** M17 cells were transduced with Ad-COX-2 and incubated with Chromofungin (1 mM) with oligomeric Aβ_42_ (5 μM). (**A**) mRNA expression of hTERT and TERF2; (**B**) Telomerase activity; (**C**) The mRNA levels of p21; (**D**) Cellular senescence was examined using SA-β-gal staining at day 14 (*n* = 6, ^**^P < 0.01 vs. vehicle group; ^††^*P* < 0.05, 0.01 vs. Aβ_42_ group; ^※, ※※^*P* < 0.05, 0.01 vs. Aβ_42_+Chromofungin group).

To confirm the role of COX-2, cells were transduced with Ad-COX-2 and incubated with Chromofungin (1 mM) with oligomeric Aβ_42_ (5 μM). The decreased hTERT level observed in Aβ_42_-stimulated M17 neuronal cells was markedly increased by Chromofungin, which was remarkably reversed by COX-2 overexpression. Moreover, the elevated TERF2 level observed in Aβ_42_-stimulated M17 neuronal cells was notably repressed by Chromofungin, which was sharply reversed by COX-2 overexpression (Figure 7A). Furthermore, the telomerase activity in Aβ_42_-stimulated M17 neuronal cells was declined from 28.6 to 17.9 IU/L, then elevated to 24.7 IU/L by Chromofungin. After COX-2 overexpression, the telomerase activity was reversed to 16.1 IU/L (Figure 7B). Furthermore, the increased p21 level (Figure 7C) after 24-h incubation and elevated proportion of SA-β-gal staining cells after 14-day incubation in Aβ_42_-stimulated M17 neuronal cells were sharply reduced by Chromofungin, which was remarkably reversed by COX-2 overexpression (Figure 7D).

## DISCUSSION

Neuronal cell senescence refers to the gradual degeneration and decline of neuronal cell function and structure. With increasing age, the functionality of neuronal cells begins to decrease, including reduced synaptic transmission efficiency, metabolic activity, and cellular protective mechanisms. This senescence process may be associated with various factors, including genetics, environment, lifestyle, and diseases [22]. The regulatory pathways of neuronal cell senescence are complex and involve the participation of multiple molecules and signaling pathways. Among them, two main regulatory pathways are OS and the inflammatory response. OS refers to the production of excessive ROS within cells, surpassing the capacity of the antioxidant defense system, leading to cell damage and senescence [23]. Inflammatory response is caused by the excessive activation of inflammatory factors, which can induce cell death and inflammatory damage, thereby accelerating neuronal cell senescence [24]. There is a close relationship between neuronal cell senescence and AD. AD is a neurodegenerative disorder characterized by the senescence and death of neuronal cells in the brain [11]. Research has shown that there are common regulatory pathways and molecular mechanisms between neuronal cell senescence and AD. For example, OS and inflammatory response play important roles in the pathogenesis of AD, which is related to the regulatory pathways of neuronal cell senescence [25]. Additionally, protein aggregation and abnormal metabolism are observed in the brains of AD patients, which are also related to neuronal cell senescence [26]. Herein, in line with data presented by Wang [26], declined telomerase activity and enhanced cell senescence were observed in Aβ_42_-stimulated M17 neuronal cells, which were markedly alleviated by Chromofungin, suggesting an anti-senescent property of Chromofungin. Moreover, the OS state in Aβ_42_-stimulated M17 neuronal cells was sharply activated and was remarkably suppressed by Chromofungin, implying that the anti-senescent property of Chromofungin might be correlated to impeded OS. These effects are similar to Klotho in Aβ_42_-stimulated SH-SY5Y cells reported by Sedighi [27].

The main function of hTERT in cells is to maintain telomere length and delay the process of cellular senescence. Telomeres are repetitive DNA sequences at the ends of chromosomes, and they shorten with each cell division. When telomeres become too short, cells enter a state of senescence or undergo apoptosis. During the process of cellular senescence, the expression of hTERT typically decreases, resulting in telomere shortening and cells entering a state of senescence [28, 29]. On the other hand, TERF2 expression usually increases during the process of cellular senescence [30]. P21, also known as CDKN1A, plays a key role in cellular senescence, which is also known as CDKN1A. When cells are damaged or subjected to other stress stimuli, the expression of p21 is upregulated, thereby inhibiting cell proliferation and division. Such cell cycle inhibition helps to maintain cellular stability and promote the entry of cells into a state of senescence [31, 32]. Herein, the influence of Chromofungin on hTERT, TERF2, and p21 expressions further confirmed the anti-senescent property of Chromofungin.

COX-2 participates in pathological processes such as inflammation and injury. Its main function is to catalyze the conversion of arachidonic acid into prostaglandins, such as PGE_2_, thereby mediating inflammatory responses and pain perception [33, 34]. The relationship between COX-2 and cellular senescence has been reported. COX-2 inhibitors have been reported to alleviate the cellular senescence in nonalcoholic fatty liver disease (NAFLD) mice [35] and the upregulation of COX-2 can trigger OS, which causes DNA damage and lipid peroxidation of the cell membrane, thereby accelerating the process of cellular senescence [36]. COX-2 siRNAs slightly reduced H_2_O_2_-induced SA-β-gal activities in hTERT-immortalized human dermal or prostatic fibroblasts (HDFs), suggesting that COX-2 mediates cellular senescence [37]. Correspondingly, cisplatin-induced SA-β-gal activities were decreased in COX-2-overexpressing CNE1 cancer cells and increased in COX-2(−/−) murine fibroblasts, indicating that COX-2 inhibits cellular senescence [38]. Furthermore, the upregulation of COX-2 can trigger inflammatory responses, thereby accelerating the process of cellular senescence [39]. Herein, COX-2/PGE_2_ axis was sharply activated in Aβ_42_-stimulated M17 neuronal cells, which was markedly repressed by Chromofungin, suggesting that Chromofungin might exert the anti-senescent effect by repressing COX-2. Moreover, the suppressive effect of Chromofungin against Aβ_42_-induced cellular senescence in M17 neuronal cells was rescued by COX-2 overexpression, further confirming that COX-2 was a mediator in the anti-senescent function of Chromofungin. In the future work, the anti-AD property will be of Chromofungin studied and identified utilizing an animal model.

Collectively, Chromofungin protects neurons from Aβ_42_-induced senescence by inhibiting COX-2. These findings suggest that Chromofungin may serve as a potential agent for the treatment of AD.
